# Multi-omics association analysis reveals interactions between the oropharyngeal microbiome and the metabolome in pediatric patients with influenza A virus pneumonia

**DOI:** 10.3389/fcimb.2022.1011254

**Published:** 2022-10-28

**Authors:** Qian Hu, Baiming Liu, Yanqun Fan, Yuejie Zheng, Feiqiu Wen, Uet Yu, Wenjian Wang

**Affiliations:** ^1^ Department of Respiratory Diseases, Shenzhen Children’s Hospital, Shenzhen, China; ^2^ Department of Trans-omics Research, Biotree Metabolomics Technology Research Center, Shanghai, China; ^3^ Department of Hematology and Oncology, Shenzhen Children’s Hospital, Shenzhen, China

**Keywords:** influenza A viruses, pneumonia, pediatric, oropharyngeal, microbiota, metabolites

## Abstract

Children are at high risk for influenza A virus (IAV) infections, which can develop into severe illnesses. However, little is known about interactions between the microbiome and respiratory tract metabolites and their impact on the development of IAV pneumonia in children. Using a combination of liquid chromatography tandem mass spectrometry (LC-MS/MS) and 16S rRNA gene sequencing, we analyzed the composition and metabolic profile of the oropharyngeal microbiota in 49 pediatric patients with IAV pneumonia and 42 age-matched healthy children. The results indicate that compared to healthy children, children with IAV pneumonia exhibited significant changes in the oropharyngeal macrobiotic structure (*p =* 0.001), and significantly lower microbial abundance and diversity (*p <* 0.05). These changes came with significant disturbances in the levels of oropharyngeal metabolites. Intergroup differences were observed in 204 metabolites mapped to 36 metabolic pathways. Significantly higher levels of sphingolipid (sphinganine and phytosphingosine) and propanoate (propionic acid and succinic acid) metabolism were observed in patients with IAV pneumonia than in healthy controls. Using Spearman’s rank-correlation analysis, correlations between IAV pneumonia-associated discriminatory microbial genera and metabolites were evaluated. The results indicate significant correlations and consistency in variation trends between *Streptococcus* and three sphingolipid metabolites (phytosphingosine, sphinganine, and sphingosine). Besides these three sphingolipid metabolites, the sphinganine-to-sphingosine ratio and the joint analysis of the three metabolites indicated remarkable diagnostic efficacy in children with IAV pneumonia. This study confirmed significant changes in the characteristics and metabolic profile of the oropharyngeal microbiome in pediatric patients with IAV pneumonia, with high synergy between the two factors. Oropharyngeal sphingolipid metabolites may serve as potential diagnostic biomarkers of IAV pneumonia in children.

## Introduction

Influenza A virus (IAV) is a common pathogen causing respiratory tract infections in children. Seasonal influenza epidemics are caused by the H1N1 and H3N2 IAV subtypes (Fouchier et al., 2005). Most infected children show mild symptoms, but this infection can lead to severe and life-threatening lung disease (Iuliano et al., 2018; Ratre et al., 2020). In children with IAV infection, secondary bacterial infections can lead to more severe diseases, such as pneumonia, pulmonary edema, and lung abscess, and morbidity and mortality are also significantly higher in children with combined *Streptococcus* pneumoniae infections compared to influenza alone (Dawood et al., 2014; Hsing et al., 2022). The potential mechanisms underlying IAV infection have not been fully explored. Early detection can considerably improve disease management and overall survival of children. New evidence suggests that the pathogenesis of IAV infection involves complicated interactions among viral invasion processes, the respiratory tract microbiome, and host mucosal immune responses (Lim et al., 2016; Gounder and Boon, 2019). During IAV infection, microbiota colonizing different ecological niches are closely associated with disease severity, duration, and prognosis (Söderholm et al., 2016). The oropharyngeal mucosa is colonized by various microorganisms, such as *Streptococcus* spp., *Neisseria* spp., and *Rothschild* spp., which maintain a dynamic balance with the lower respiratory tract to ensure the physical health of the host. The oropharyngeal mucosa is an integral part of the mucosal epithelial barrier, which isolates numerous bacteria, but is a notable site of virus entrance into the body, proliferation, and transmission (Man et al., 2017; Shannon et al., 2021).

To date, little is known about the mechanisms underlying the impact of the microbiota and its metabolites on inflammatory responses and mucosal immune function in pediatric patients. A metabolite is an intermediate or final product of a cellular regulatory process. Therefore, the level of a macrobiotic metabolite can reflect the cellular biochemical activities associated with infections (Jaurila et al., 2020). Direct metabolite testing has become a valuable method for identifying biomarkers of various diseases and exploring potential pathogeneses. Metabolite analyses can distinguish active infections from latent ones, thus addressing the shortcomings of available diagnostic tests, such as polymerase chain reaction (PCR), in disease diagnosis and treatment (Bowler et al., 2017; Zurfluh et al., 2018). Previous studies have reported that infections can induce changes in *in vivo* metabolites and affect the microbiotic structure, exacerbating respiratory diseases (Gu et al., 2019; Mendez et al., 2019). The microbiota can participate in host physiological and pathological processes by converting nutrients provided by the host into metabolites (Anand et al., 2016). Through the direct or indirect stimulation of the host immune system, IAV can disrupt cellular metabolic pathways by obtaining the components necessary for its self-replication from host cells (Smallwood et al., 2017).

However, most studies focus on mono-omics, cell culture, or animal models (Wen et al., 2018; Gierse et al., 2021). Therefore, the interactions between the microbiome and the respiratory tract and their correlations with susceptibility to IAV infection and disease severity in children remain unclear. Therefore, our objective was to identify changes in the oropharyngeal microbiome and metabolite profile of pediatric patients and to analyze the association between the two omics. Our findings may elucidate the etiology of IAV-related pneumonia in children and provide more accessible and valuable information for early risk prediction.

## Materials and methods

### Study participants and sample collection

Study participants comprised 49 children with IAV pneumonia hospitalized at Shenzhen Children’s Hospital (Shenzhen, China) and 42 age- and sex-matched healthy controls (<16 years of age) who underwent a health examination from January 2018 to January 2020. The age difference between the two groups was maintained at <6 months to reduce potential confounding differences in the metabolome and microbiome between different age groups. Participants in both study groups were Han Chinese, with similar dietary habits and geographical proximity (Shenzhen, China). Participants in the IAV pneumonia group met the following criteria: 1) epidemiological history, clinical symptoms, and radiological signs of acute IAV pneumonia were present; 2) IAV positive status was confirmed using multiplex kits for the detection of 13 respiratory pathogens (PCR capillary electrophoresis fragment analysis; Haiers Gene Technology Co., Ltd., Ningbo, China); and 3) the pediatric patient joined the clinical pathway for pneumonia immediately after hospital admission and received unified and standardized treatment. Furthermore, pediatric patients were included in the severe pneumonia group based on their clinical symptoms and whether they were admitted to the Pediatric Intensive Care Unit (PICU). Exclusion criteria included: 1) The presence of any medical conditions including acute upper and lower respiratory tract infections (rhinitis, tonsillitis, bronchitis, and pneumonia, as well as severe odontogenic, oral, and maxillofacial infections), within one month before enrollment, may affect the oropharyngeal microbiota and metabolome. Chronic diseases include asthma and cystic fibrosis. 2) Nutritional status was judged by experienced pediatricians according to 2006 child growth standard (WHO and Multicentre Growth Reference Study Group, 2006), using weight-for-age growth curves directly for children under five years and body mass index (BMI) for children over five years (de Onis et al., 2007). BMI (kg/m^2^) = weight/height^2^. The 5th percentile ≤ BMI < 85th percentile is defined as normal weight. Malnutrition and obesity were excluded.3)Use of drugs that affect the microbiome and metabolome, such as immunosuppressants, probiotics, traditional Chinese medicine, and glucocorticoids, within 1 month before enrollment. 4) Essential data were missing because of refusal of laboratory tests after admission. 5) Patients with delayed consent for enrollment (>24 hours after hospitalization) (Stewart et al., 2017). The study flowchart, from enrollment to analysis, is shown in **Supplementary Figure 1**. The Medical Ethics Committee of Shenzhen Children’s Hospital of China Medical University approved this study (registration number: 202009202). All parents of children who took part in the study provided us with their written, informed consent.

On the day of the health examination, oropharyngeal specimens were collected from healthy controls and patients with IAV pneumonia sterile swabs (155C, COPAN, Murrieta, CA, USA) within 24 h after hospital admission (Hogan et al., 2021; Ma et al., 2021). We collected samples in the morning, and participants refrained from brushing their teeth 12 hours before sampling, rinsed or drank water two hours before sampling to remove oral debris, followed by a two-hour fast to reduce the impact on the oropharyngeal microbiota and metabolome. A sterile tongue depressor was inserted to fully expose the deep pharynx. Then the swab was inserted into the pharynx and rotated twice against the posterior pharyngeal wall or the pharyngeal-palatal arch, avoiding contact with other areas such as the tongue, uvula, and gingiva. An experienced pediatrician or nurse performed all procedures. The oropharyngeal swab samples were immediately put on ice and sent to a biological specimen bank, where they were stored at -80°C until further analysis. Unused swabs were used as blank controls to assess contamination during the experiment.

#### Oropharyngeal DNA extraction for microbiome analysis

Using the power soil DNA isolation kit (Mo Bio Laboratories, Carlsbad, CA, USA) and following the manufacturer’s instructions, total genomic DNA was isolated from oropharyngeal swab samples (Wen et al., 2018). A Thermo NanoDrop 2000 spectrophotometer (Thermo Fisher Scientific, New York, NY, USA) was used to measure the DNA concentration. 2% agarose gel electrophoresis was used to confirm the DNA’s integrity and fragment size. Total DNA was stored in an elution buffer at -80°C until PCR sequencing was performed.

### 16S ribosomal RNA gene sequencing with high throughput

The V3-V4 region of the 16S ribosomal RNA (rRNA) gene was amplified using the PCR primers (341F: 5′-CCTACGGGNGGCWGCAG-3′ and 805R: 5′-GACTACHVGGGTATCTAATCC-3′). The PCR products were measured using Qubit (Invitrogen Ltd., Carlsbad, CA, USA) after being purified with AMPure XT beads (Beckman Coulter Genomics, Danvers, MA, USA). The Illumina sequencing Library Quantification Kit (Kapa Biosciences, Woburn, MA, USA) and an Agilent 2100 Bioanalyzer (Agilent Technologies, Inc., Santa Clara, CA, USA) were used to confirm the amplicon library before up-sequencing the qualifying libraries. We performed 2 × 250 bp double-end sequencing on the NovaSeq 6000 platform (Illumina, San Diego, CA, USA), using the NovaSeq 6000 SP Reagent Kit (500 cycles); all was done following the manufacturer’s instructions. The assembled miseq sequence was submitted to NCBI’s open-access sequence to read the archive.

#### Sequencing data analysis

Pair-end reads obtained after sequencing were divided into samples for data separation based on their unique barcode information. In addition, the primer sequence and barcode were removed. The raw reads were quality filtered according to fqtrim (v0.94) (Magoč and Salzberg, 2011), and the double-ended sequences that passed the primary quality screening were pairwise linked based on overlapping bases using FLASH (v1.2.8). The Vsearch software (v2.3.4) was used to identify and reject chimeric sequences. The feature abundance table and the feature sequence of the amplicon sequence variants (ASVs) were obtained using a divisive amplicon denoising algorithm 2 (DADA2). Bioinformatic analysis of the oropharyngeal microbiome was performed using QIIME 2 software. The annotation was performed using the SILVA database (Release 138) and the NT-16S database based on the ASV feature sequence (Quast et al., 2013). The abundance of each species was determined according to the ASV abundance table. The confidence threshold for the annotation was >0.7.

### Preparation of oropharyngeal samples for metabolomics analysis

Ultra-high performance liquid chromatography mass spectrometry (UHPLC-MS/MS) was used to determine the metabolite composition of oropharyngeal swab samples. Oropharyngeal swabs were transferred to Eppendorf (EP) tubes; 1000 ml of the extraction solution was added to the EP tube in an acetonitrile: methanol: water ratio of 2:2:1 (V:V:V). The tubes were then vortexed for 30 s and placed in an ice-water bath for 30 min of sonication. After removing the swabs, the supernatant was stored at -40°C for 1 h. The samples were then centrifuged at 4°C and 13,800 ×*g* for 15 min, and the supernatant obtained was transferred to a new injection vial for onboard detection.

### Liquid chromatography mass spectrometry/mass spectrometry analysis

In both positive and negative-ion modes, all oropharyngeal samples were subjected to metabolite separation using a UHPLC system (Vanquish, Thermo Fisher Scientific). The target compounds were separated using a Waters ACQUITY UPLC BEH Amide (2.1 mm × 100 mm, 1.7 µm) liquid chromatography column. Mobile phase A was an aqueous phase with a pH of 9.75. It contained 25 mmol/L of ammonium acetate and 25 mmol/L of ammonia hydroxide, whereas mobile phase B comprised acetonitrile. The temperature of the sample tray was 4°C, and the injection volume was 2μL.

The organic phase was injected into the column at 30°C. The elution gradients were set to 95% B, 0–0.5 min; 95–65% B, 0.5–7.0 min; 65–40% B, 7.0–8.0 min; 40% B, 8.0–9.0 min; 40–95% B, 9.0–9.1 min; and 95% B, 9.1–12.0 min. The data was gathered using Xcalibur (Thermo Fisher Scientific) on the Orbitrap Exploris 120 mass spectrometer, which can collect primary and secondary mass spectrometry data in the information-dependent acquisition mode. Other conditions for the electrospray ionization source were established as follows: capillary temperature, 320°C; collision energy, 10/30/60 in NCE mode; MS/MS resolution, 15,000; full MS resolution, 60,000; auxiliary gas flow rate, 15 Arb; and sheath gas flow rate, 50 Arb. The spray voltage was set to 3.8 and -3.4 kV for the positive-ion and negative-ion modes, respectively.

### Data analysis

The raw data were transformed into the mzXML format using the ProteoWizard software. The R package, XCMS (v3.8.2), was used to perform peak identification, peak alignment, peak extraction, and integration. The algorithm scoring cutoff value was set to 0.3. The online Human Metabolome Database and Kyoto Encyclopedia of Genes and Genomes (KEGG) database were used to compare molecular weight data (m/z) for the identification of metabolites. The deviation values were filtered according to the coefficient of variation, the missing values in the data were filled by one-half of the minimum value, the individual peaks were filtered, and the peak area of the internal standard in each sample was normalized. In over 50% of the samples, missing ions were considered low-mass ions and were removed. Finally, 9820 peaks were retained; the raw data were uploaded to the MetaboLights website.

### Statistical analyses

Data were statistically analyzed using R (v3.6.3, R Foundation for Statistical Computing, Vienna, Austria) and SPSS (v22.0, Statistical Product and Service Solutions, IBM, Chicago, IL, USA). Data are presented as mean ± standard deviation, and the Mann-Whitney U test was performed to compare two independent samples having a non-normal distribution. The count data are expressed as the number of cases or percentages (%), and the Chi-square test was used to compare groups. The R vegan package was used to perform the permutational multivariate analysis of variance (PERMANOVA), and 1,000 permutations were used to calculate the adonis p-value (Zhou et al., 2020; Frau et al., 2021). Spearman rank-correlation analysis was used to determine the associations between the microbiome and metabolites. The final data set from the LC-MS/MS analysis was imported into the SIMCA 16.0.2 software package (Sartorius Stedim Data Analytics AB, Umea, Sweden). The importance of the variable in the projection (VIP) of the first principal component of each metabolite was calculated by building an orthogonal partial least squares discriminant analysis model (OPLS-DA). Seven-fold cross-validation was used to obtain R^2^ and Q^2^ to assess model validity. The Mann-Whitney U test was used to determine differences between the two groups; differences defined as *p <* 0.05 and VIP > 1 were considered significant. The Benjamini-Hochberg correction method was used to adjust the P values.

## Results

### Clinical characteristics

Children <6 years accounted for 89.8% (44/49 cases) of the patients in the IAV pneumonia group, with children aged 3–6 years accounting for most cases (53.1%, 26/49 cases). No significant differences were observed between the age, gender, exposure to cigarettes at home, antibiotics use before sampling, and child delivery method of the two groups (*p* > 0.05) (**Supplementary Table 1**). Furthermore, PERMANOVA was performed to adjust for these and found that IAV pneumonia was the main factor contributing to the difference in microbiota and metabolome between the two groups (permutated *p* = 0.001). Moreover, there was no statistically significant difference between antibiotics on microbiota or metabolome (permutated *p* = 0.774 and *p* = 0.352, respectively). The average time from the onset of influenza symptoms to hospital admission was 7.08 ± 3.13 days (**Supplementary Table 1**). Seventeen pediatric patients were admitted to the PICU for further treatment, as required for their disease conditions, and classified as patients with severe pneumonia.

### Oropharyngeal microbiota profile

Oropharyngeal swab samples collected from the 49 pediatric patients with IAV pneumonia and 42 healthy individuals were subjected to 16S rRNA sequencing. Two samples (FG20 and HG20) were removed due to a failure to detect the microbiota, leaving 89 samples for the final analysis. A total of 5,049,593 high-quality sequences were obtained (average: 58,385 sequences per sample; range: 49,637.50–64,538.50). After species annotation was performed, 4,133 usable ASVs were obtained (**Supplementary Table 2**; average, 176 ASVs per sample; range: 103.5–225.0), and the data of these ASVs were subjected to a structural analysis of the oropharyngeal microbiome. The rarefaction curves for the two groups of samples leveled or plateaued, indicating that the sequencing depth was adequate for identifying the features of most bacteria in the samples and the subsequent structural analysis (**Supplementary Figure 2A–E**). The rank abundance distribution curves suggested that the IAV pneumonia group had a significant decrease in abundance and microbiota imbalance compared to the healthy group (**Supplementary Figure 2F**).

**Figure 1:**
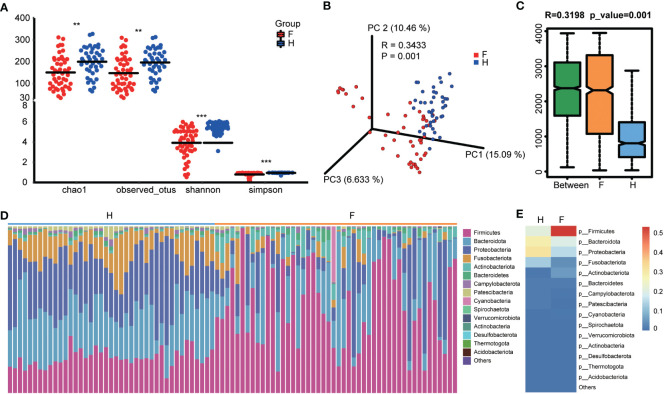
Diversity and structural analysis of the oropharyngeal microbiota. **(A)** Alpha diversity differences between the two groups were observed using the Simpson index, Shannon index, observed species, and chao1. **p < 0.05; ***p < 0.001; F, IAV pneumonia group (red); H, healthy group (blue). **(B)** PCoA plot based on the Bray–Curtis distance matrix reflects the difference in bacterial structure between the two groups. F, IAV pneumonia group (red); H, healthy group (blue). **(C)** Through unweighted unifrac analysis of similarity, we discovered that the difference between groups was significantly greater than within groups. F, IAV pneumonia group (yellow); H, healthy group (blue). **(D)** Relative abundances of the top 15 phyla in each group. F, IAV pneumonia group (yellow); H, healthy group (blue). **(E)** The heatmap reflects the similarities and differences in phylum composition between the two groups in terms of color gradients. IAV, influenza A virus.

The sequences were analyzed to estimate the alpha and beta diversity and measure variations in microbial diversity between the two groups. Alpha diversity analysis indicated significant intergroup differences in the Shannon index (3.92 ± 1.45 versus 5.39 ± 0.56, *p <* 0.001), observed species (147.75 ± 72.55 versus 196.53 ± 63.37, *p <* 0.001), chao1 (150.77 ± 73.40 versus 199.99 ± 65.85, *p <* 0.001), and Simpson index (0.79 ± 0.22 versus 0.94 ± 0.05, *p <* 0.001), suggesting significantly lower abundance and diversity of the oropharyngeal microbiota in the IAV pneumonia group than in the healthy volunteer group (**Figure 1A**). Beta diversity describes the intergroup differences in species. The beta diversity analysis indicated substantial changes in the composition and richness of the oropharyngeal microbial community between the two groups (Bray–Curtis *p =* 0.001; **Figure 1B**). Based on an analysis of similarity, we found that the differences between groups were significantly greater than those within groups. We observed a higher beta diversity in the oropharyngeal microbiota of children with IAV pneumonia, indicating that the microbiota structure of the IAV pneumonia group was more heterogeneous than that of the control group **Figure 1C**).

### Influenza A pneumonia-associated changes in the oropharyngeal microbiota

The species abundance table for each taxonomic level was obtained based on the ASV annotations and abundance tables for the analysis and comparison of the species composition of the two groups. The IAV pneumonia group exhibited significantly differential abundances of 12 bacterial phylum, 12 classes, 33 orders, 46 families, 63 genera, 63 species, and 838 individual ASVs (**Supplementary Table 3**). At the phylum level, bacteria belonging to 11 phyla were identified in the healthy volunteer group, whereas 14 phyla were identified in the IAV pneumonia group. Firmicutes, Bacteroidota, Proteobacteria, Fusobacteriota, and Actinobacteria were the dominant bacterial phyla in both groups (**Figure 1D**). However, intergroup differences were observed in the proportions of the oropharyngeal microbiota represented by these predominant phyla. The differences in the proportion of Firmicutes were the most notable (**Figure 1E**). In the IAV pneumonia group, Firmicutes represented 53.04% of bacteria, whereas in the healthy volunteer group, Firmicutes accounted for 21.18%. In contrast, Bacteroidota and Proteobacteria represented higher proportions in the healthy volunteer group (28.85% vs. 19.57% and 31.77% vs. 15.22%, respectively).

**Figure 2:**
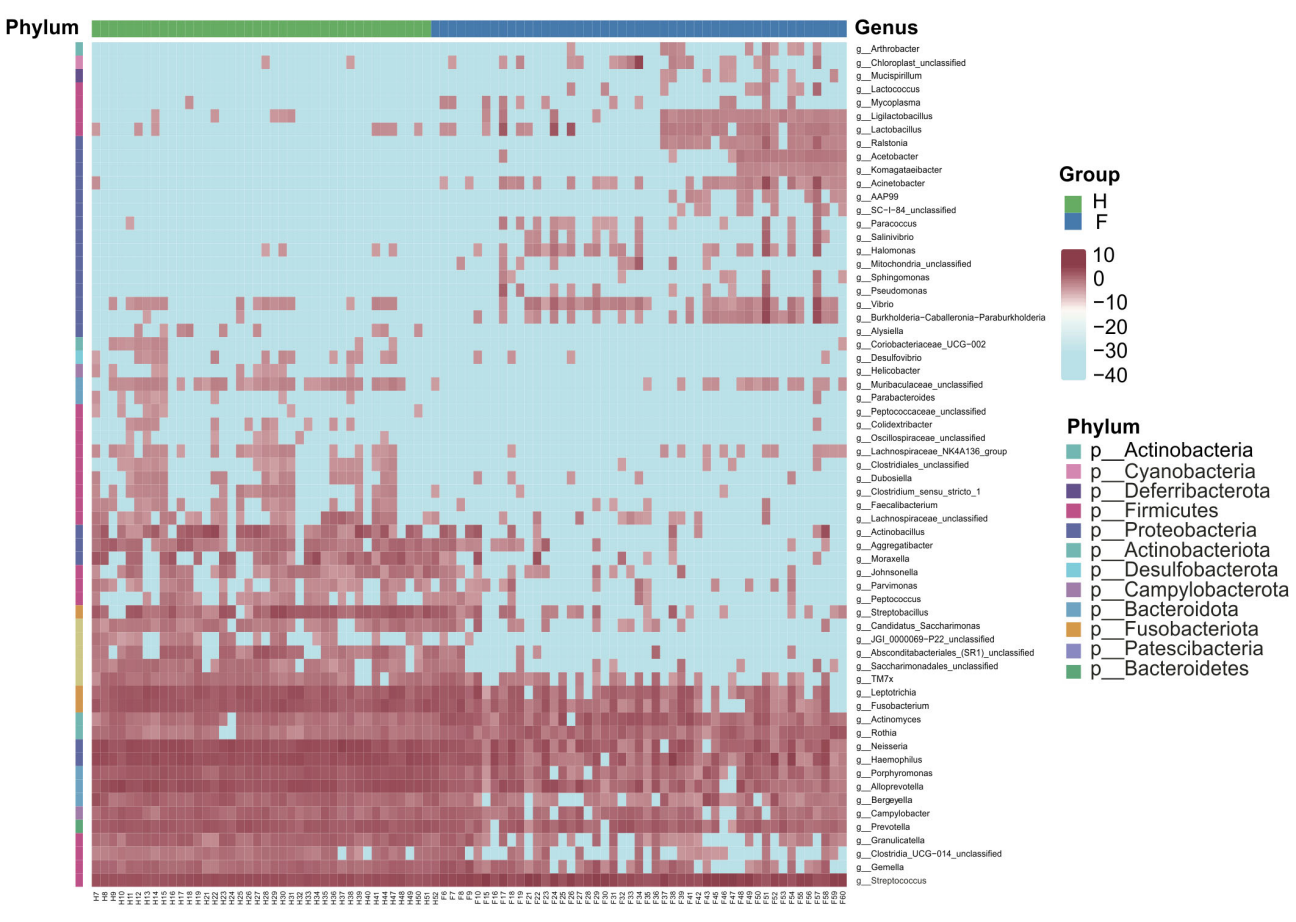
The two groups have similarities and differences in composition at the phyla and genera levels. The data after Z-transformation showed the abundance of genera, which was lower in the blue section and higher in the red section. F, IAV pneumonia group (blue); H, healthy group (green). IAV, influenza A virus.

Further evaluation of differences between the microbiota at different taxonomic levels revealed that Firmicutes (*p <* 0.001) and Actinobacteriota (*p <* 0.001) were more abundant in the IAV pneumonia group than in the healthy volunteer group at the phylum level. However, the abundance of Proteobacteria (*p <* 0.001), Fusobacteriota (*p <* 0.001), and Bacteroidota (*p <* 0.001) was significantly higher in the healthy volunteer group (**Supplementary Figure 3A**). At the genus level, there were significant intergroup differences for 63 genera (FDR *p <* 0.05); the abundance levels of *Streptococcus* (*p <* 0.001) and *Actinomyces* (*p <* 0.001) were significantly higher in the IAV pneumonia group, whereas the abundance levels of *Haemophilus* (*p <* 0.001), *Neisseria* (*p <* 0.001), *Alloprevotella* (*p <* 0.001), and *Leptotrichia* (*p <* 0.001) were significantly higher in the healthy volunteer group (**Supplementary Figure 3B**). The heatmap of the relative abundance of the 63 genera, listed in **Supplementary Table 4**, is shown in **Figure 2**. We also analyzed PICU admission; however, intergroup differences were not observed at any taxonomic level (all FDR *>* 0.05).

**Figure 3:**
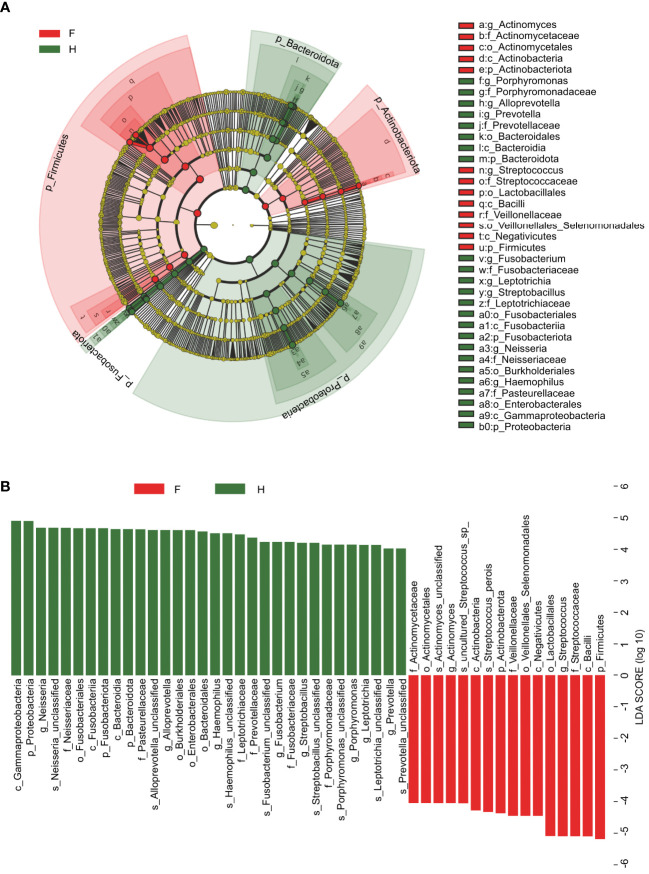
Linear discriminant analysis (LDA) and LDA effect size. **(A)** The cladogram visually indicates the differential species in each level in each group. F, IAV for abstract pneumonia group (red); H, healthy group (green). **(B)** Histogram of LDA scores for different abundant genera between groups H and (F) Red, enriched in the F group; Green, enriched in the H group. F, IAV pneumonia group; H, healthy group; IAV, influenza A virus.

We generated a cladogram using linear discriminant analysis (LDA) effect size analysis to identify the oropharynx-specific microbiota associated with pediatric IAV pneumonia by visually presenting all strata of differential species in the two groups (**Figure 3A**). Significant differences were observed between the oropharyngeal microbiota of the two groups in 48 ASVs (LDA > 4), with relatively high abundances of *Actinomyces*, *Streptococcus*, *Lactobacillales*, and *Veillonellaceae* (all LDA scores log_10_ > 4) in the IAV pneumonia group. In contrast, the abundances of *Bacteroidales*, *Porphyromonas*, *Haemophilus*, *Neisseria*, *Streptobacillus*, and *Prevotella* were significantly higher in the healthy volunteer group (all LDA scores log_10_ > 4) than in the IAV pneumonia group. Thirty-two ASVs were enriched in the healthy volunteer group and sixteen ASVs in the IAV pneumonia group, suggesting higher abundances in the healthy volunteer group. These results indicate a lower abundance of microbiota in the IAV pneumonia group. Hence, there was a difference in the abundance of the oropharyngeal microbiota between the two groups (**Figure 3B**).

### General characteristics of the oropharyngeal metabolome in each group

We expected that the oropharyngeal microbiota in children with IAV pneumonia would partially influence oropharyngeal metabolic pathways, based on previous mono-omics studies that demonstrated a high correlation between IAV infection and the microbiome and metabolome (Ohno et al., 2020; Gierse et al., 2021). Therefore, LC-MS/MS-based untargeted metabolomics were used to identify and quantify 591 metabolites from 91 oropharyngeal samples from the two groups (**Supplementary Table 5**).

### Discriminatory oropharyngeal metabolites

**Figure 4:**
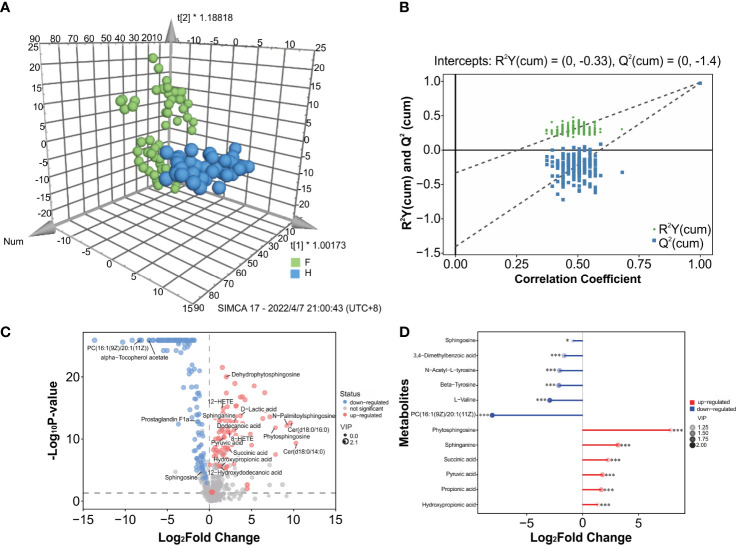
Metabolite differences between the IAV pneumonia group F and the healthy group H. **(A)** The OPLS-DA 3D plot of the oropharyngeal samples from both groups (*p <* 0.001) are shown. F, IAV pneumonia group (green circle); H, healthy group (blue circle). **(B)** The OPLS-DA permutation test indicates good model robustness, with no overfitting (n = 200). **(C)** Volcano plots showing differences in oropharyngeal metabolites between children with IAV pneumonia and healthy controls: upregulated metabolites (red circles), downregulated metabolites (blue circles). **(D)** Important discriminatory metabolites are displayed on the matchstick diagram. *p < 0.05; ***p < 0.001. The abscissa shows the log-transformed change multiple, and the dot color depth represents the VIP value size. IAV, influenza A virus.

Significant variations in metabolic phenotypes were detected between the two groups in the OPLS-DA model, implying that patients with IAV pneumonia had a distinct metabolic profile (R^2^X (cum) = 0.357, R^2^Y (cum) = 0.985, Q^2^ (cum) = 0.974, *p <* 0.001) (**Figure 4A**). The permutation test revealed no overfitting and that the OPLS-DA model was remarkably robust(n = 200; **Figure 4B**). In order to see the general distribution, we displayed the results of the differential metabolite screening as volcano plots. The IAV pneumonia group had relatively high abundances of 86 metabolites and relatively low abundances of 118 metabolites (*p <* 0.05; VIP > 1; **Supplementary Table 6**, **Figure 4C**). Sphingosine, L-valine, 3,4-dimethylbenzoic acid, and N-acetyl-L-tyrosine were significantly reduced in the IAV pneumonia group, according to a matchstick graph of differential metabolites. In contrast, phytosphingosine, sphinganine, succinic acid, pyruvic acid, propionic acid, and hydroxypropionic acid increased significantly in the IAV pneumonia group (**Figure 4D**).

### Revealing discriminatory metabolites by different analytical methods

**Figure 5:**
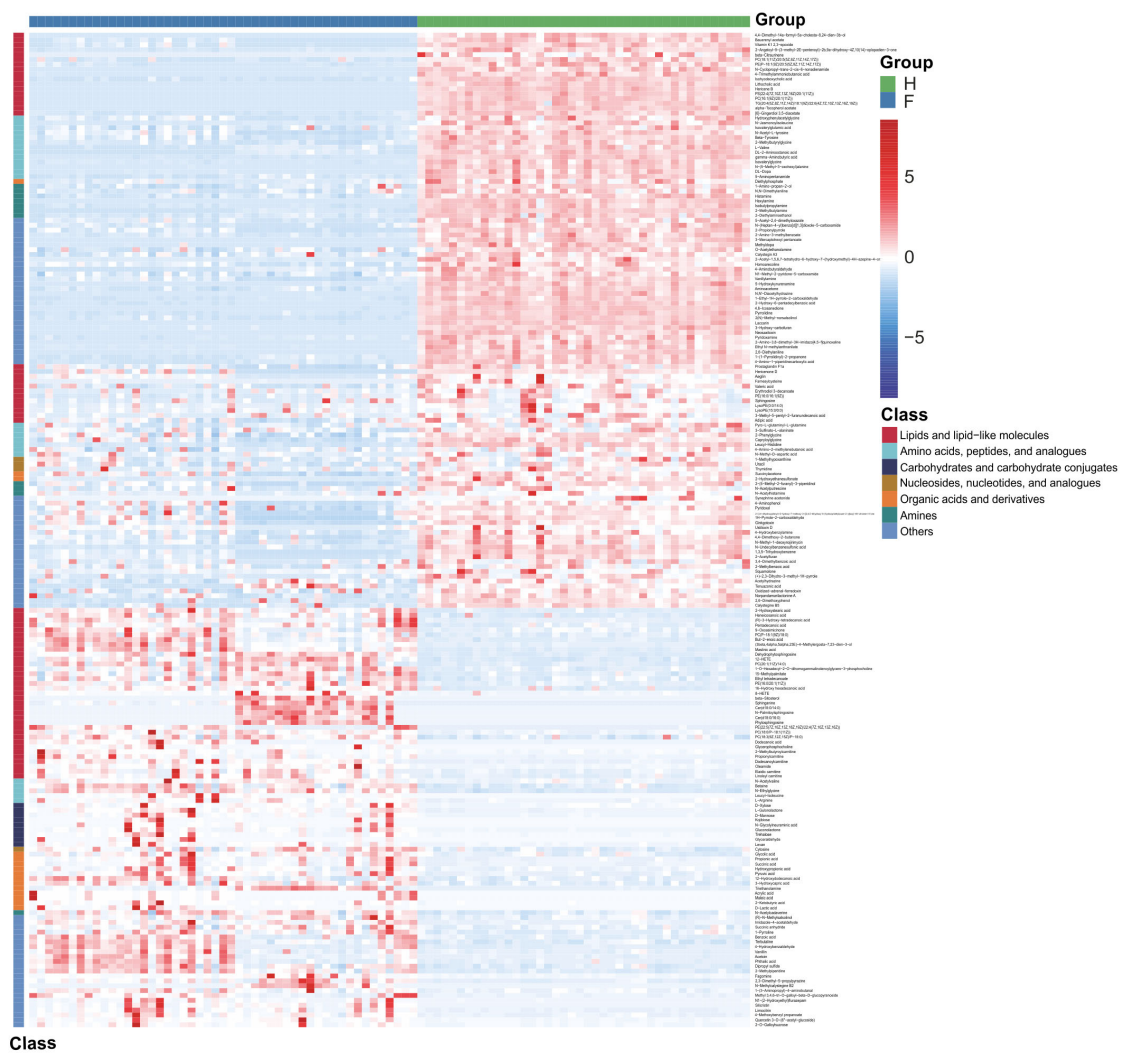
Hierarchical cluster analysis (HCA) was performed using quantitative values of differential metabolites in two groups. The horizontal coordinates represent the grouping, and the vertical coordinates represent the differential metabolites in the group. Red indicates high expression, whereas blue indicates low expression. F, IAV pneumonia group (blue); H, healthy group (green). IAV, influenza A virus.

**Figure 6:**
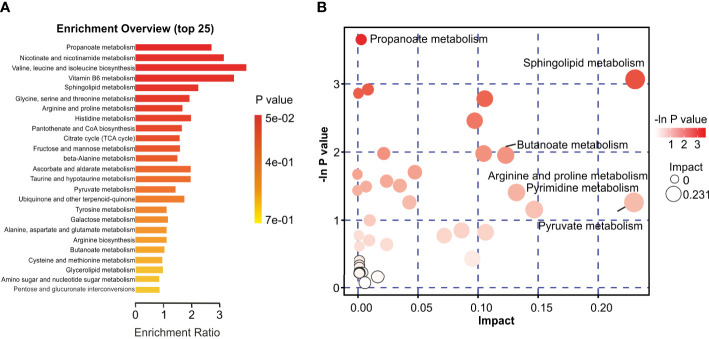
Characteristics of the oropharyngeal metabolome in IAV pneumonia and healthy volunteer groups. **(A)** Differential Pathway enrichment analysis based on differential metabolites between the two groups. **(B)** The crucial pathway with the highest correlation with metabolite differences between groups F and H was identified following metabolic pathway analysis of differential metabolites. In the bubble chart, each bubble represents a metabolic pathway. The X-axis and bubble size indicate the size of the impact value of the pathway; the Y-axis and bubble color indicate the p-value (-ln_p_) of the enrichment analysis. F, IAV pneumonia group; H, healthy group; IAV, influenza A virus.

The identified discriminatory metabolites were usually functionally similar and biologically complementary to each other. Hierarchical cluster analysis (HCA) revealed that in the healthy volunteer group, abundant metabolites were glycerophosphocholine {PC[16:1(9Z)/20:1(11Z)]}, amino acid metabolites (beta-tyrosine, L-valine, and N-acetyl-L-tyrosine), and lipids and lipid-like molecules (bauerenyl acetate, isohyodeoxycholic acid, lithocholic acid, and alpha-tocopherol acetate). In contrast, the IAV pneumonia group displayed relatively high levels of amino acid metabolites (L-arginine, leucyl-isoleucine, and N-ethylglycine), sphingolipid metabolites (sphinganine and phytosphingosine), propanoate metabolites (propionic acid, succinic acid, and hydroxypropionic acid), and alpha hydroxy acids and derivatives (D-lactic acid). Significant changes in the oropharyngeal metabolome occurred in the disease group, with lipid metabolism being the most pronounced. Lipid metabolites represented 31.4% of the 204 discriminatory metabolites (64/204; **Figure 5**). Total oropharyngeal metabolites were mapped to 47 KEGG metabolic pathways *via* KEGG annotation and classification of metabolites. The enriched metabolites were associated with propanoate metabolism (4 metabolites), sphingolipid metabolism (5 metabolites), ABC transporters (23 metabolites), tyrosine metabolism (10 metabolites), and nicotinate and nicotinamide metabolism (9 metabolites). We identified the differential metabolic pathways involved in IAV pneumonia by KEGG annotation. Compared to healthy controls, metabolic pathway analysis of patients with IAV pneumonia revealed 36 pathways with differently abundant metabolites (**Figure 6A**). The results of the metabolic pathway analysis are shown in the bubble plots in **Figure 6B**, where the differences in propanoate and sphingolipid metabolism are significant (*p <* 0.05; **Supplementary Tables 7, 8** **)**.

**Figure 7:**
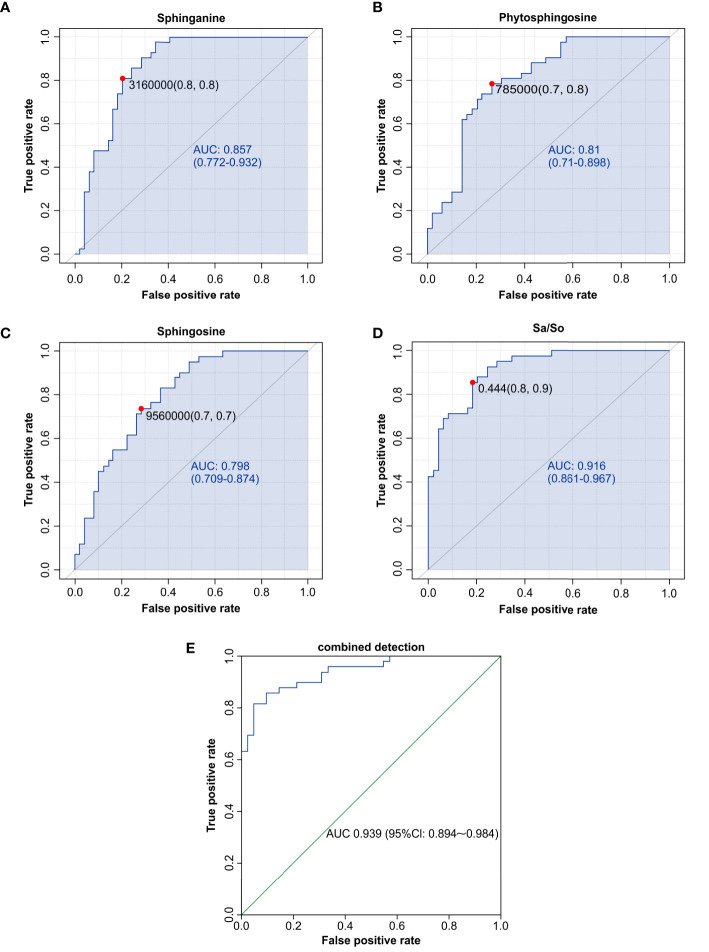
Sphingolipid metabolites, such as sphinganine **(A)**, phytosphingosine **(B)**, sphingosine **(C)**, the Sa/So ratio **(D)**, and the combination of multiple indicators **(E)**, were discovered to have high AUCs by ROC analysis and could be used as biomarkers for influenza-induced pneumonia in children. AUC, area under the curve; ROC, receiver operating characteristic.

Based on previous results and the known importance of the role of sphingolipid metabolites in viral infectious diseases, we defined discriminatory metabolites in the sphingolipid metabolic pathway as potential biomarkers for IAV pneumonia (Avota et al., 2021). Sphinganine (area under curve [AUC], 0.857; *p <* 0.001), phytosphingosine (AUC, 0.810; *p <* 0.001), and sphingosine (AUC, 0.798; *p <* 0.001) were significantly related to IAV pneumonia. A previous study revealed that an elevated sphinganine-to-sphingosine (S_a_/S_o_) ratio indicated a disturbance in sphingolipid biosynthesis, which was correlated with the host’s inflammatory response (Antonissen et al., 2015). Our investigation indicated that the S_a_/S_o_ ratio (AUC, 0.916; *p <* 0.001) was also associated substantially with IAV pneumonia. Furthermore, analysis of the combined diagnosis using sphingosine, phytosphingosine, and sphinganine yielded an AUC of 0.939 (*p <* 0.001), indicating a close correlation between the three metabolites and IAV pneumonia in children. Therefore, oropharyngeal sphingolipid metabolites could serve as another powerful diagnostic tool and biomarkers for IAV pneumonia in children (**Figure 7**).

### Multi-omics analysis method reveals the association between the oropharyngeal microbiome and metabolites for the two groups

**Figure 8:**
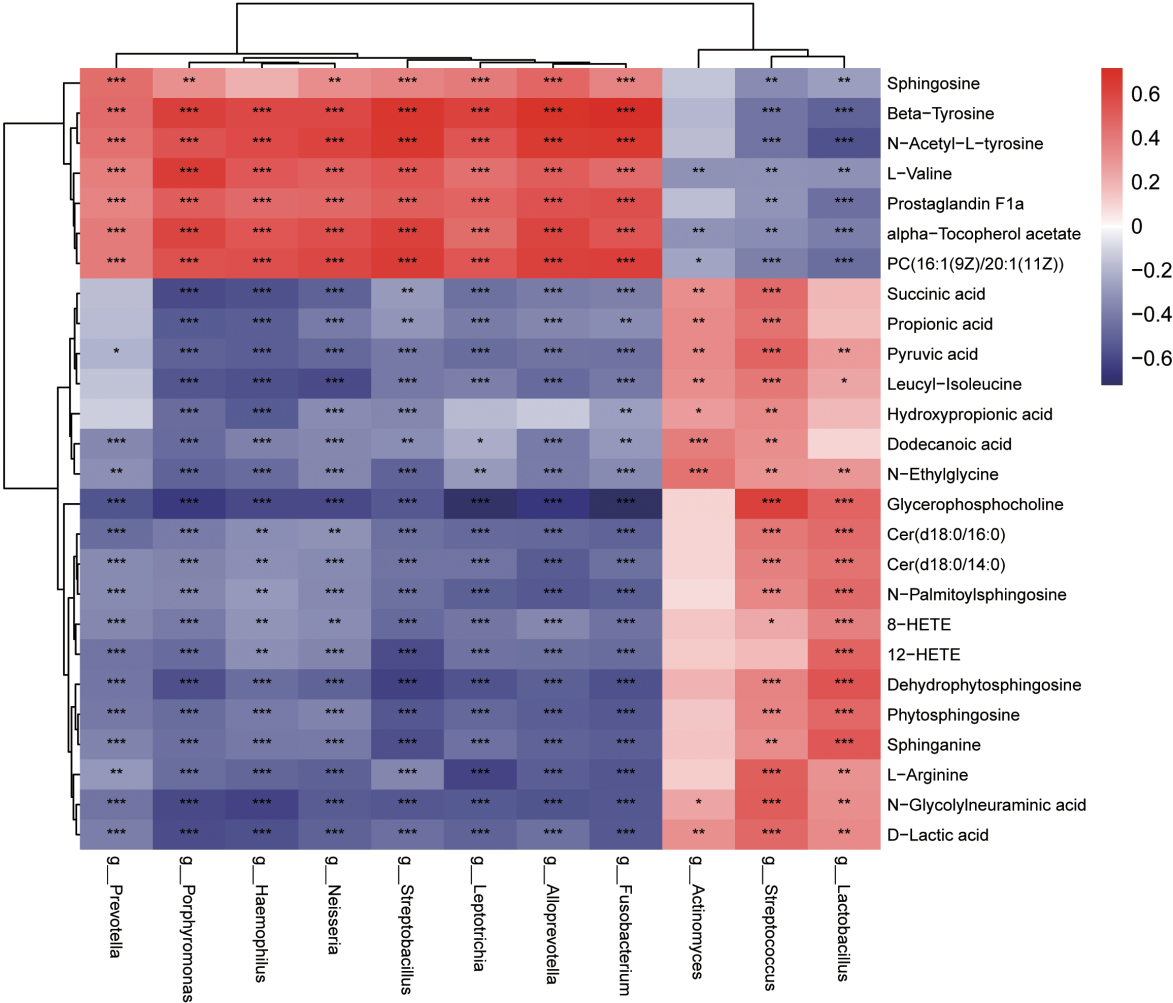
Heatmaps indicate the Spearman rank-correlation coefficients for the relative abundance of differential oropharyngeal microbiota at the genus level and differential metabolites in children with IAV pneumonia and healthy controls. Positive correlation is depicted using red, whereas negative correlation is depicted using blue. *p < 0.05; **p < 0.01; ***p < 0.001; IAV, influenza A virus.

Based on the results of our study, 10 genera (LEfSe LDA > 4and *p <*0.05) and 26 metabolites differentially expressed (VIP >1 and *p <*0.05) were used to verify the changes in the correlation between genera and metabolites in the oropharynx of children with IAV pneumonia. Most discriminatory oropharyngeal metabolites were significantly correlated with differential microbiomes and the trends in their variations were consistent (|r| >0.3 and *p <*0.05). The significantly increased abundance of the genus, *Streptococcus*, was positively correlated with the significantly increased metabolites in the IAV pneumonia group, including sphinganine (r = 0.3255, *p =* 0.0019), phytosphingosine (r = 0.3560, *p =* 0.0007), N-palmitoylsphingosine (r = 0.3476, *p =* 0.0009), and L-arginine (r = 0.5021, *p <* 0.001), and negatively correlated with the significantly less abundant metabolites in the IAV pneumonia group, including β−tyrosine (r = -0.4305, *p <* 0.001) and PC (16:1(9Z)/20:1(11Z)) (r = -0.3867, *p =* 0.0002). Furthermore, the higher abundance of the genus, *Actinomyces*, in the IAV pneumonia group was positively correlated with significantly more abundant metabolites in this group, including succinic acid (r = 0.3232, *p =* 0.0020) and propionic acid (r = 0.3416, *p =* 0.0011). However, there were no significant correlations between *Actinomyces* and phytosphingosine, and between sphinganine and N-palmitoylsphingosine. Moreover, the genera enriched in the healthy volunteer group, such as *Haemophilus*, were positively correlated with the most abundant metabolites from this group, including β-tyrosine (r = 0.5890, *p <* 0.001), L-valine (r = 0.5274, *p <* 0.001), and prostaglandin F1a (r = 0.4594, *p <* 0.001), and negatively correlated with the least abundant metabolites in this group, including sphinganine (r = -0.4163, *p <* 0.001) and phytosphingosine (r = -0.4082, *p <* 0.001; **Figure 8**, **Supplementary Table 9**).

## Discussion

This study confirmed that IAV pneumonia in pediatric patients is associated with changes in the structure of the oropharyngeal microbiota and its metabolites and that these two factors are significantly correlated and consistent in terms of variation trends. Therefore, we combined two omics to identify potential biomarkers associated with IAV pneumonia in children. There are four types of influenza viruses, among which IAV infection (especially H1N1) causes more severe illness in patients (Drews et al., 2019; Gaitonde et al., 2019). Viruses have applied multiple strategies to evade the host’s immune response, including an antigenic shift to evade vaccine protection. Furthermore, first line anti-influenza drugs are limited in terms of time of administration and drug resistance; this requires the development of drugs that target the metabolic mechanisms (Hurt, 2014; Zumla et al., 2016). The oropharynx is a space shared by the external environment, the respiratory system, and the digestive system, and serves as a major source of the microbiome in the lower respiratory tract, explains the striking similarity between the composition of the microbiota in the oropharynx and that in the lower respiratory tract (Sahin-Yilmaz and Naclerio, 2011; Bassis et al., 2015). The oropharyngeal mucosal epithelium is a vital defense barrier of the human body that can protect directly or indirectly against invading pathogens. With technological advancements, studies of oropharyngeal and nasopharyngeal microbiomes and metabolomes have become feasible (Marangoni et al., 2020; Ma et al., 2021). Pediatric patients and their family members relatively comply with oropharyngeal swab sampling due to its non-invasive nature (Huang et al., 2020).

Our study showed significant changes in the composition of the oropharyngeal microbiota of pediatric patients with IAV pneumonia, such as a significant decrease in the abundance and diversity of the microbiota and an increase in the heterogeneity of the bacterial community structure, which is consistent with previous findings. (Hanada et al., 2018). The disease group showed higher Firmicutes at the phylum level, while the healthy group showed higher Bacteroidota and Proteobacteria. Studies on the oropharyngeal microbiota in adult patients with IAV pneumonia have also revealed significantly higher abundances of Firmicutes and Proteobacteria, consistent with this study (Leung et al., 2013). At the genus level, the abundances of *Streptococcus* and *Actinomyces* were significantly higher in the IAV pneumonia group. Previous studies suggest that specific microbial communities in the respiratory mucosa directly or indirectly influence the host’s defense against viral infections. The microbiota can influence viral infections through various mechanisms, including the promotion of viral replication, such as stabilization of viral particles, modulation of the host immune response to viral infections, and the promotion of adenovirus reactivation by metabolites of bacterial short-chain fatty acids (SCFAs), which cause recurrent respiratory infections in children (Wang et al., 2021; Wirusanti et al., 2022).In childhood, IAV infections that are more dependent on innate immune responses, the immune system preferentially uses T helper 2 cell (Th-2) responses associated with γ δ T cells, which may be less favorable than the Th-1 antiviral immune response in adults; also, this is influenced to some extent by the gut microbiome (Sakleshpur and Steed, 2022). Furthermore, viral infection-induced host antiviral immune responses can disrupt the microbiota structure and function (Hanada et al., 2018). Viral infections usually increase glucose uptake and fermentation processes, thus altering the composition of the microbiota and clinical symptoms (Shibata et al., 2020). Studies have shown that IAV inhibits bacterial-induced interleukin-1β (IL-1β) production and impairs host defense against bacterial infection. IL-10 production by regulatory T (T Reg) cells has the potential to induce susceptibility to secondary bacterial infections, and serum levels of IL-1β and IL-10 are elevated in children secondary to Streptococcus pyogenes infection one week after IAV infection, while decreasing with symptom improvement, which seems to indicate that IAV infection and secondary Streptococcus infection also enhance pulmonary toxicity in children, but this needs to be confirmed by further studies (Bedoya et al., 2013; Ochi et al., 2018). According to these investigations, bacterial-viral interactions play an important role in disease development (Bai et al., 2022; Mirzaei et al., 2022).

Interestingly, the study discovered that the IAV pneumonia group had much greater levels of *Streptococcus* and *Actinomycetes* at the genus level. However, in previous studies, children infected with IAV show a higher abundance of *Streptococcus* in the nasopharynx and lower abundances of *Streptococcus* and *Neisseria* in the oropharynx during the early phase of infection (Wen et al., 2018). Using a decision tree evaluation by random forest analysis, Zhou et al. (2020) demonstrated that elevated Ralstonia and Acidobacteria in oropharyngeal samples could better distinguish between healthy groups and respiratory infections groups such as IAV and Mycoplasma pneumoniae infections, consistent with our study. Existing studies suggested that nasopharyngeal secretions can drip down the posterior part of the nasal cavity into the oropharynx or lungs, resulting in migration of nasal microbes into the lungs. The immune barrier and the indigenous microbiota in the respiratory tract gradually eliminate nasal microbes to restore homeostasis (Gu et al., 2019). Entering virus particles can bind to the respiratory colonization microbiota and stimulate local microbiota reproduction and diffusion to the upper and lower respiratory tracts when infected with IAV; however, this follows viral infection by a few days and correlates with lower respiratory macrophage loss (Mina et al., 2015). Dynamic changes in the structure of the gut microbiota over time and an increase in the abundance of several bacterial genera, including *Acinetobacter* and *Streptococcus*, were observed on day 7 after infection in mouse models infected with influenza A (Gu et al., 2019). In children, the vulnerability to secondary bacterial superinfection often manifests one week after influenza infection, and much research has sought to understand the immunological mechanism of IAV exacerbated by bacterial superinfection (Ochi et al., 2018). In our study, the mean time to the onset of influenza symptoms was seven days at the time of admission of children with IAV pneumonia, and the patients had a significant increase in oropharyngeal *Streptococcus*. These studies suggest that the abundance of oropharyngeal *Streptococcus* in children is altered following IAV infection, which may exacerbate lung injury and the disease. However, more studies are needed to understand how changes in the microbiota in children affect different responses of the immune system to IAV infection and the mechanisms by which lung injury occurs. There is the potential to confer beneficial immunomodulatory effects through microbiological interventions and to assist in targeted clinical treatment.

Significant changes in the oropharyngeal metabolome of study participants with IAV pneumonia were observed, the most significant changes in lipid metabolism. Pathway enrichment analyses revealed significant changes in propanoate and sphingolipid metabolism. Previous studies in human lung epithelial cells and animal models revealed alterations in lipids, carbohydrates, and related molecules, nucleosides, and other metabolites following influenza virus infection, which follows the present findings (Cui et al., 2016; Tisoncik-Go et al., 2016; Tian et al., 2019). Sphingolipid metabolites are essential components of the lipid bilayer and the extracellular fluid. They serve as signaling molecules in normal cellular physiological processes and pathological inflammatory conditions (Avota et al., 2021). sphingosine, sphinganine, and phytosphingosine are categorized as long-chain sphingolipid bases and are essential in cellular apoptosis as sphingolipid structural analogs (Nagahara et al., 2005). Therefore, sphingosine, phytosphingosine, sphinganine, the S_a_/S_o_ ratio, and the combination of the three metabolites showed good diagnostic value in children with IAV pneumonia.

IAV primarily targets mucosal epithelial cells in the respiratory tract (Lee et al., 2018). Viruses must interact with cell membranes to attach and infiltrate cells as specialized intracellular pathogens. Sphingolipid metabolites perform various functions in virus-host interactions, including the promotion of virus binding and the entrance, reproduction, and release of new particles (Konan and Sanchez-Felipe, 2014; Drake et al., 2017). This study revealed a significantly lower abundance of sphingosine and significantly higher abundances of sphinganine and phytosphingosine in the IAV pneumonia group than in the healthy volunteer group. A previous study showed that phytosphingosine and sphingosine exert different levels of antimicrobial activity against several species of bacteria (Fischer et al., 2012). Adult patients with severe IAV infections also exhibit changes in the sphingolipid metabolites in their blood plasma, which is closely associated with respiratory failure and death. The bronchial mucosal epithelium acts as the host’s first line of defense against respiratory infections, and sphingosine is a component of this epithelium. Sphingosine can inhabit various bacterial species *in vivo* and *in vitro*, serving as a natural antiseptic agent in the airways (Wendt et al., 2021). Sphingosine expression levels are significantly reduced in patients with cystic fibrosis and mouse models, resulting in an increased incidence of pulmonary infections, which are ameliorated by the sphingosine pathway or by inhalation of exogenous sphingosine (Grassmé et al., 2017). In addition, sphinganine protects lung tissue from invading pathogens, and significantly elevated serum levels of sphinganine in mouse models infected with SARS-CoV-2 are correlated with disease severity (Vitner et al., 2022). As a result, oropharyngeal sphingolipid metabolites may be useful as both diagnostic and therapeutic targets for childhood IAV pneumonia. Furthermore, pediatric patients with IAV had significantly elevated levels of propionic acid and succinic acid, resulting from propanoate metabolism and lower levels of L-valine. Propionic acid induces insulin resistance and hyperinsulinemia by activating the sympathetic nervous system in mice (Tirosh et al., 2019). In addition, significantly higher serum levels of propionic acid were observed in patients with SARS-CoV-2 pneumonia, which could act as a potential biomarker of metabolic disorders related to COVID-19 (He et al., 2021). Elevated levels of succinic acid indicate an increased turn of the TCA cycle, whereas increased TCA turn rates and mitochondrial dysfunction can lead to oxidative stress in patients (Bolukbas et al., 2005; Li et al., 2015).

We also performed a multi-omics association analysis and discovered that discriminatory oropharyngeal microbiota was closely associated with discriminatory metabolites. For example, *Streptococcus* was positively correlated with sphinganine, phytosphingosine, and N-palmitoylsphingosine, suggesting that oropharyngeal microbiota and metabolites had consistent variation trends. Some investigations have discovered a strong correlation between elevated *Streptococcus* levels and the change in sphingolipid metabolites. Previous investigations on nasopharyngeal samples from infants with severe bronchiolitis found that the sphingolipid pathway is the most enriched sub-pathway positively correlated with abundance of Streptococcus (Stewart et al., 2017). In adult patients with community-acquired pneumonia, changes in *Streptococcus* in the respiratory tract were significantly correlated with pneumonia severity and were associated with changes in serum metabolites, including sphingolipid, pyruvate, and inositol phosphate (To et al., 2016). Studies indicate that the abundance of many species of *Streptococcus* has significantly increased in patients with chronic obstructive pulmonary disease (COPD). The increased abundance of the glucosyltransferase and LP_X_TG-anchored adhesion domain in *streptococcus* enrichment suggests that the ability to the stick to surfaces was essential for increased abundance (Bowerman et al., 2020). Furthermore, bioactive sphingolipids are becoming evident in the regulation of cell adhesion, migration, and invasion, with sphinganine, phytosphingosine and sphingosine modulating bacterial adhesion, which may be a key point of interaction between Streptococcus and sphingolipid metabolites (Hannun and Obeid, 2018; Cukkemane et al., 2015). Metagenomic data analysis confirmed that *Streptococcus* produces serine, the substrate for sphinganine and the fundamental building block of all sphingolipids; therefore, exogenous serine produced by *Streptococcus* can contribute to a significant increase in airway metabolism (Stewart et al., 2017).However, the mechanisms involved need to be further explored. Furthermore, in this study, Lactobacillus and D-lactate in the oropharynx of children with IAV pneumonia were significantly elevated and positively correlated. Lactobacillus-produced D-lactic acid in the oropharynx may play a vital role in inhibiting *Streptococcus* colonization and proliferation (Yildiz et al., 2020). These results demonstrate that pediatric patients with IAV pneumonia show changes in the oropharyngeal microbiota and its metabolites during acute infections and that significant correlations between the oropharyngeal microbiota and oropharyngeal metabolites can be identified. Combining these findings, we found that IAV pneumonia leads to an altered abundance of specific microbiota in the airways and alters host cell metabolism. On the one hand, the oropharyngeal microbiota can contribute to the altered severity of IAV pneumonia by modulating host cell function and metabolism (e.g., sphingolipid metabolism). However, the abundance of specific microbiota (e.g., *Streptococcus*) is altered following changes in host metabolism. Our findings should support future research into the potential processes relating these changes in the microbiota, host immune system, and airway metabolism to IAV development. These findings are highly significant for early disease prediction, evaluation, and intervention.

We adopted a multi-omics analytical approach and revealed significant changes in the oropharyngeal microbiota and metabolites, as well as significant correlations between the two factors in pediatric patients with IAV pneumonia. Oropharyngeal swabbing serves as a convenient, effective, and non-invasive sampling method that facilitates scientific evaluation. However, this study has several limitations. Because our findings are based on data collected from a single center with a relatively small sample size, more multicenter studies that use larger datasets are needed to validate our metagenomic and metabolomic findings. Furthermore, we performed comparative analyzes between pediatric patients with IAV infection only and healthy children; therefore, the generalizability of the findings of our study to pediatric patients with mixed infections is unclear. In subsequent studies, other important respiratory viruses should be included in comparative analyzes to optimize the evaluation of the available molecular diagnostic approaches. Finally, although the selection of subjects included in this study was rigorous, ethically,children with pneumonia are required to receive medication as soon as possible.There may still be various potential factors affecting the microbiome and metabolome. In a subsequent investigation, we will set up a more thorough longitudinal study to reduce confounders and dynamically track changes in the respiratory microbiome and metabolome of IAV-infected children. However, the results of this preliminary study provide important clues for understanding the respiratory microbiome and metabolome associations in children with IAV pneumonia and to explore potential predictors and more effective treatment options.

## Conclusions

This study comprehensively analyzes the mechanism of the oropharyngeal microbiota and its metabolites compared to previous mono-omics studies of IAV pneumonia among pediatric patients. Oropharyngeal samples from pediatric patients with IAV pneumonia can be successfully differentiated from those of healthy children using LC-MS/MS-based untargeted metabolomics and high throughput 16S rRNA gene sequencing-based microbiome analysis. Pediatric patients with IAV pneumonia had significantly lower abundance and diversity of the oropharyngeal microbiota than healthy children, with significant changes in the abundance of bacterial species such as *Streptococcus*, *Rothia*, and *Haemophilus*. Furthermore, significant intergroup differences in oropharyngeal metabolites were observed. Among them, the sphingolipid metabolites, sphingosine, sphinganine, and phytosphingosine were identified as important discriminatory oropharyngeal metabolites. These three metabolites, the S_a_/S_o_ ratio, and the combination of these three metabolites showed high diagnostic efficacy in pediatric patients with IAV pneumonia. The characteristic changes in the oropharyngeal microbiota and metabolites indicate they can serve as efficient and non-invasive diagnostic biomarkers, and even therapeutic targets for pediatric patients with IAV pneumonia. More long-term confirmatory studies are required in a larger patient population across different geographic regions and ethnic groups to validate these hypotheses.

## Data availability statement

The datasets presented in this study can be found in online repositories. The names of the repository/repositories and accession number(s) can be found in the article/**Supplementary Material**.

## Ethics statement

The studies involving human participants were reviewed and approved by The Medical Ethics Committee of Shenzhen Children’s Hospital, China Medical University (registration number: 202009202). Written informed consent to participate in this study was provided by the participants’ legal guardian/next of kin.

## Author contributions

Conceptualization: QH, FW, and WW. Methodology: QH and UY. Formal analysis: QH and YF. Data curation: BL and QH. Software: YF. Writing-original draft preparation: QH and YF. Writing-review and editing: YZ, UY, and WW. All authors contributed to the article and approved the submitted version.

## Funding

This study was supported by the Shenzhen Fundamental Research Program (JCYJ20190809170007587), Shenzhen Fund for Guangdong Provincial High-level Clinical Key Specialties (SZGSP012), Shenzhen Key Medical Discipline Construction Fund (SZXK032) and the Shenzhen Science and Technology Innovation Commission (RCBS20200714114858018).

## Acknowledgments

We thank the recruited children and their parents who participated in the research. We thank Editage (www.editage.cn) for English language editing.

## Conflict of interest

The authors declare that the research was conducted in the absence of any commercial or financial relationships that could be construed as a potential conflict of interest.

## Publisher’s note

All claims expressed in this article are solely those of the authors and do not necessarily represent those of their affiliated organizations, or those of the publisher, the editors and the reviewers. Any product that may be evaluated in this article, or claim that may be made by its manufacturer, is not guaranteed or endorsed by the publisher.
