# Supplementary material for: Multi-omics association analysis reveals interactions between the oropharyngeal microbiome and the metabolome in pediatric patients with influenza A virus pneumonia
Source: Front Cell Infect Microbiol. 2022 Oct 28;12:1011254. doi: 10.3389/fcimb.2022.1011254 (PMC9651038; doi:10.3389/fcimb.2022.1011254)
Supplement: Supplementary file 1 [file DataSheet_1.docx]

Supplementary Material

## Supplementary Figures


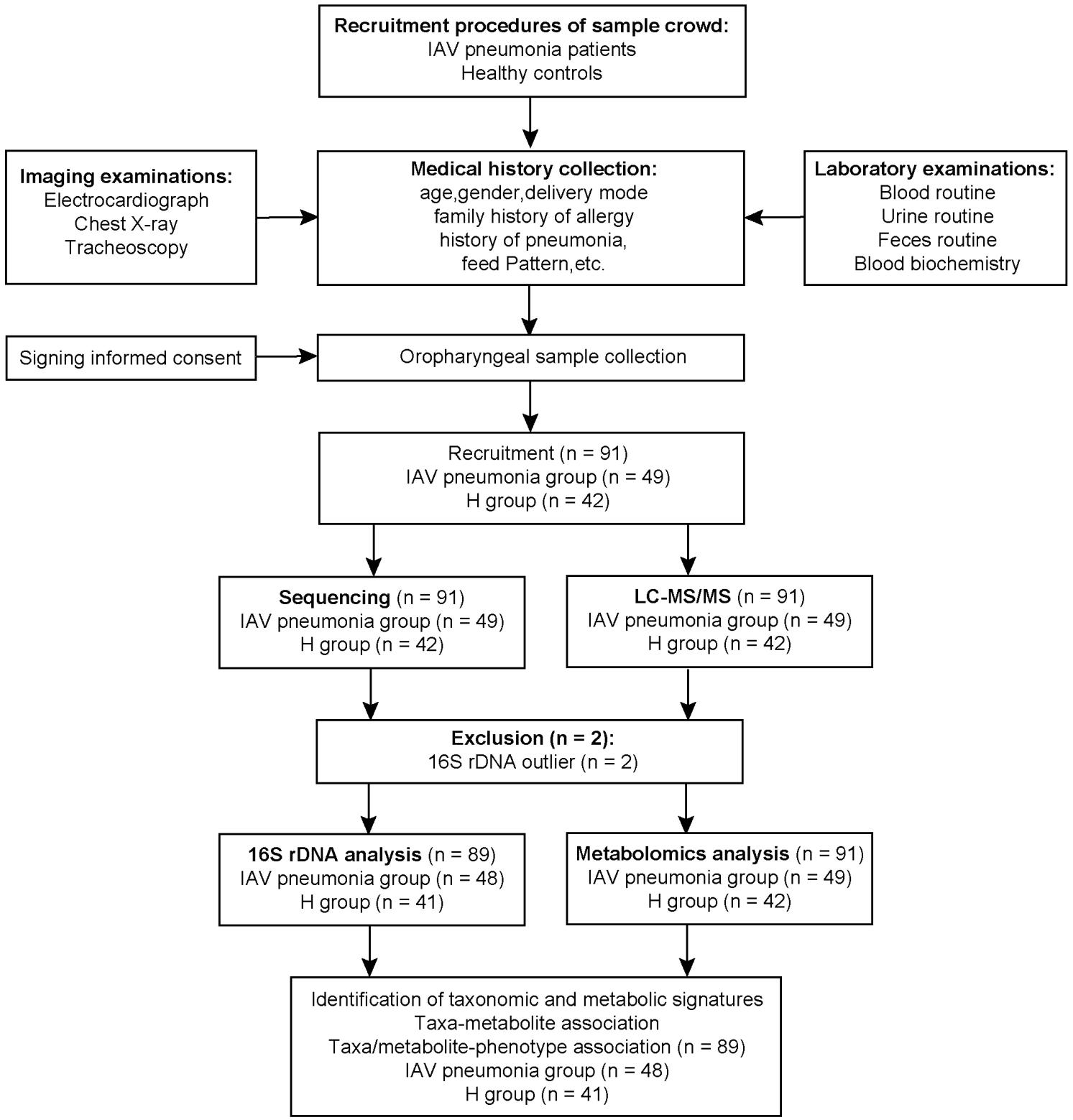


## Supplementary Figure 1. Flowchart of the inclusion and exclusion criteria and data analysis process for patients with influenza A (IAV) pneumonia (Group F) and healthy controls (Group H).


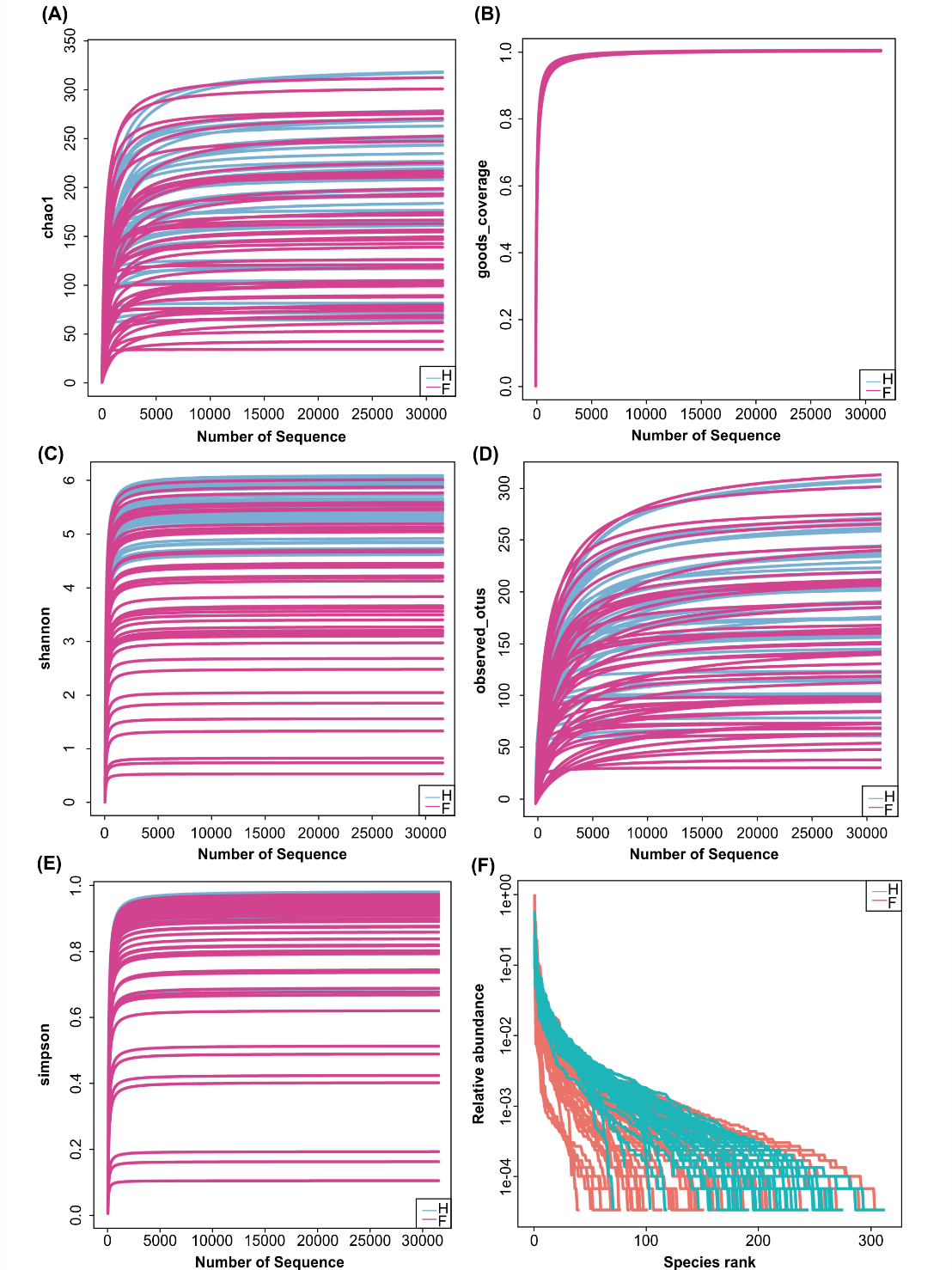


**Supplementary Figure 2.** Relative bacterial richness and evenness analyses. (A) The Chao1 index estimates the number of ASVs in the samples. (B) Coverage refers to microbial coverage, and its value is proportional to the sample sequence detection rate, indicating sufficient sequencing depth. (C) The Shannon index estimates the microbial diversity index in samples. (D) The rarefaction curves for the two groups of samples levels out or plateaus, indicating that the sequencing depth was adequate. (E) The Simpson index estimates the index of microbial diversity in samples. (F) Rank abundance distribution curve provides a direct view of species richness and evenness between groups. F, IAV pneumonia group (red); H, healthy group (blue). IAV, influenza A virus; ASV, amplicon sequence variant.


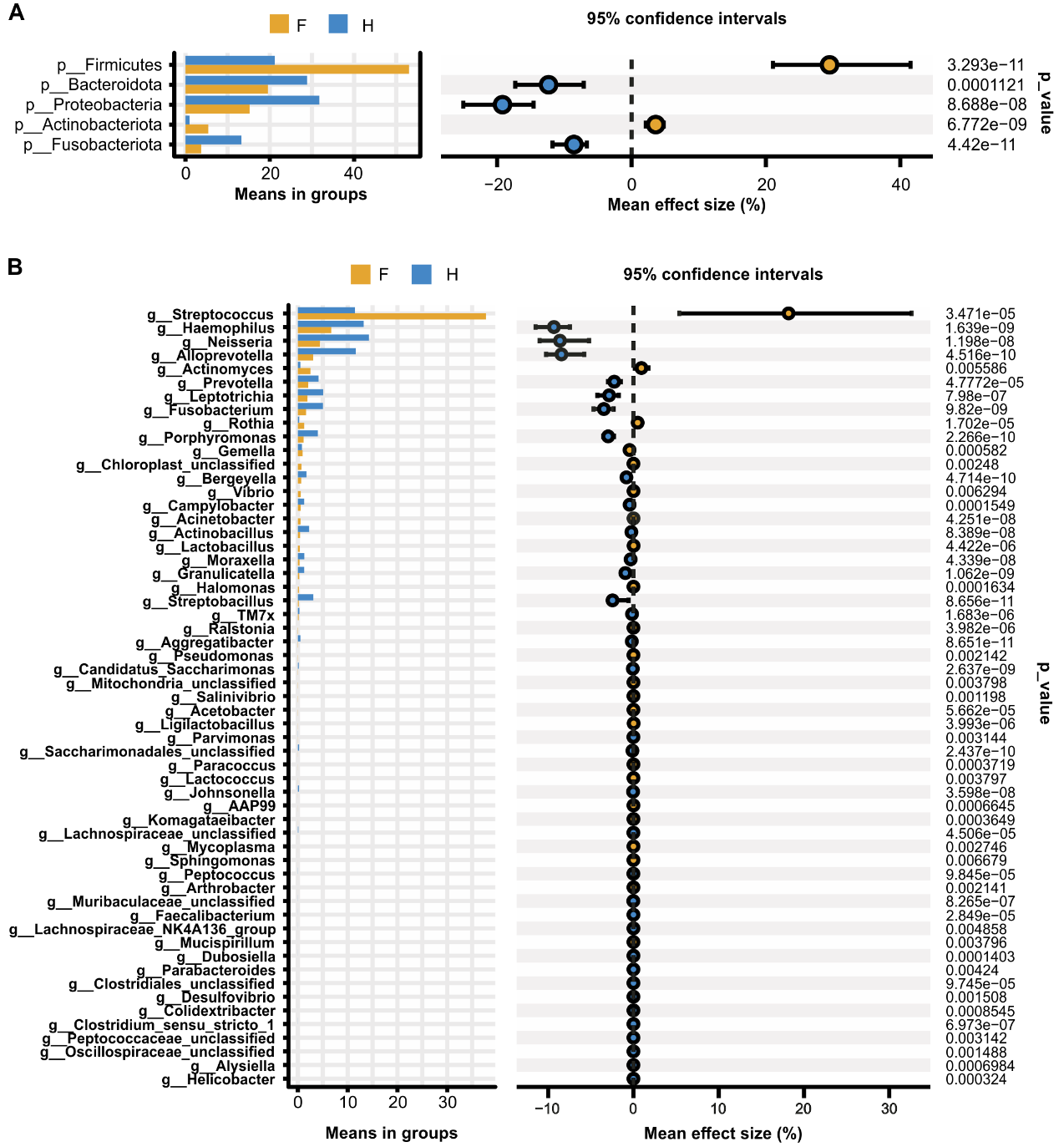


**Supplementary Figure 3.** Altered oropharyngeal microbiota structure at the phylum and genus levels associated with IAV pneumonia in children. (A) Relative abundance of the microbial composition, with significant changes at the phylum level assessed using the Wilcoxon test. (B) Relative abundance of the microbial composition, with significant changes at the genus level assessed using the Wilcoxon test. F, IAV pneumonia group (yellow); H, healthy group (blue). IAV, influenza A virus.

## Supplementary Tables

**Supplementary Table 1.** Clinicopathological factors in pediatric patients with influenza A virus pneumonia and healthy controls.

**Supplementary Table 2.** Microbiota taxonomic assignment based on the amplicon sequence variant feature table derived from exact sequencing variants. H, Healthy controls group; F, influenza A virus pneumonia group.

**Supplementary Table 3.** 16S rRNA high-throughput gene sequencing differences between groups H and F at various taxonomic levels of the microbiota. H, Healthy controls group; F, influenza A virus pneumonia group.

**Supplementary Table 4.** Heatmap analysis of 63 differential microbiota in groups F and H at the genus level based on 16S rRNA high-throughput gene sequencing. H, Healthy controls group; F, influenza A virus pneumonia group.

**Supplementary Table 5.** Liquid chromatography-tandem mass spectrometry analysis was used to identify oropharyngeal metabolite abundance in groups H and F. H, Healthy controls group; F, influenza A virus pneumonia group.

**Supplementary Table 6.** Differences in oropharyngeal metabolites between groups H and F (liquid chromatography-tandem mass spectrometry-based metabolomics dataset with fold change (FC) and Mann-Whitney U test based p-values and variable importance prediction (VIP) cores; p < 0.05; VIP > 1). H, Healthy controls group; F, influenza A virus pneumonia group.

**Supplementary Table 7.** Metabolite IDs in the Human Metabolome Database (HMDB) and Kyoto Encyclopedia of Genes and Genomes (KEGG) database for oropharyngeal differential metabolites in groups F and H. H, Healthy controls group; F, influenza A virus pneumonia group.

**Supplementary Table 8:** Results of differential oropharyngeal metabolite metabolic pathway analyses in groups F and H. H, Healthy controls group; F, influenza A virus pneumonia group.

**Supplementary Table 9.** Spearman's correlation (r) values for differential oropharyngeal metabolites and microbiota in groups F and H. H, Healthy controls group; F, influenza A virus pneumonia group.
